# Embedding of lamellar hole-associated epiretinal proliferation combined with internal limiting membrane inversion for the treatment of lamellar macular hole: a case report

**DOI:** 10.1186/s12886-018-0926-8

**Published:** 2018-09-24

**Authors:** Yusuke Shiode, Yuki Morizane, Kosuke Takahashi, Shuhei Kimura, Mio Hosokawa, Masayuki Hirano, Shinichiro Doi, Shinji Toshima, Mika Hosogi, Atsushi Fujiwara, Fumio Shiraga

**Affiliations:** Department of Ophthalmology, Dentistry and Pharmaceutical Sciences, Okayama University Graduate School of Medicine, 2-5-1 Shikata-cho Kita-ku, Okayama City, Okayama 700-8558 Japan

**Keywords:** Epiretinal membrane, Internal limiting membrane, Lamellar macular hole, Lamellar hole-associated epiretinal proliferation

## Abstract

**Background:**

We recently reported that lamellar macular hole (LMH) with lamellar hole-associated epiretinal proliferation (LHEP) can be effectively treated by embedding the LHEP into the retinal cleavage to improve foveal contour and visual acuity. Here, we report a case of LMH with LHEP for which we performed embedding of the LHEP combined with internal limiting membrane (ILM) inversion. We then evaluated the effects of this surgery on macular morphology and visual functions.

**Case presentation:**

A 62-year-old man presented with visual disturbance (20/29) and metamorphopsia in his right eye. B-scan optical coherence tomography (OCT) imaging revealed the presence of both partial-thickness defect of the macula with degenerative retinal cleavage and LHEP at the surface of the retina. En face OCT imaging showed the absence of retinal fold. We performed phacoemulsification with intraocular lens implantation, vitrectomy, embedding of LHEP into the retinal cleavage, and ILM inversion. Three months after the surgery, both foveal contour and visual acuity (20/20) were improved and metamorphopsia was reduced.

**Conclusion:**

Embedding of the LHEP combined with ILM inversion may be an effective treatment for LMH with LHEP.

**Electronic supplementary material:**

The online version of this article (10.1186/s12886-018-0926-8) contains supplementary material, which is available to authorized users.

## Background

Although an international classification of lamellar macular hole (LMH) based on B-scan images has been reported [1], the definition and classification of LMH has not yet reached a universal consensus because progressing optical coherence tomography (OCT) technology has revealed large morphological diversity of LMHs [2–8]. Recently, Govetto et al. [5] proposed that LMH can be classified into two types, tractional and degenerative, based on the presence or absence of retinal traction involvement. In support of this, our group combined radial B-scan OCT imaging and en face OCT imaging and observed that the pathology of degenerative LMH shows less involvement of retinal traction than the pathology of tractional LMH [9]. The difference in the pathologies of these LMH types suggests that differential treatment plans are necessary on the basis of retinal traction involvement. For tractional LMH, it is effective to remove the epiretinal membrane (ERM) and internal limiting membrane (ILM) in order to release retinal traction. However, the same treatment is unlikely to improve the pathology of degenerative LMH because of the lack of retinal traction involvement. Indeed, it has been reported that removal of the ERM and the ILM to treat degenerative LMH does not result in improved visual acuity or macular contour, and, furthermore, this surgery does result in an increased risk of macular hole formation [10–12]. Therefore, an effective surgical treatment method for degenerative LMH is needed.

One of the features of degenerative LMH is the high probability of a yellow atypical ERM called a lamellar hole-associated epiretinal proliferation (LHEP) at the macula [2–4]. Histologically, LHEP is mainly composed of glial cells of the retina [12, 13]. Furthermore, observations during surgery for degenerative LMH have shown that that LHEP, unlike typical ERM, connects to the retina at the edge of the foveal aperture of the LMH [14]. These findings indicate that LHEP is composed of one of the major retinal glial cells, Müller cells, which have proliferated and migrated on the retinal surface with macular pigment [14].

To address the need for an effective surgical treatment for degenerative LMH, we previously proposed a surgical treatment method in which the LHEP is embedded in the retinal cleavage of the degenerative LMH. We found that this LMH embedding technique effectively improves both macular morphology and visual acuity [14]. Since glial cells proliferate and migrate to the site of nerve injury and contribute to nerve healing by producing trophic factors and growth factors [15–18], the therapeutic mechanism of this procedure is probably due to the Müller cells within the embedded LHEP. Recently, using an experimental monkey model for large macular hole, we performed an ILM inversion technique and showed that the ILM, which is the basement membrane of Müller cells, was able to facilitate glial cell proliferation and migration as well as the expression of neurotrophic factors and growth factors from glial cells [19]. This technique thus lead to an acceleration of the wound healing processes at the macula [20]. Based on these findings, we hypothesized that the ILM might be able to facilitate the therapeutic process of the LHEP embedding technique for the treatment of degenerative LMH. In this report, we describe a case of degenerative LMH for which we performed the LHEP embedding technique combined with ILM flap inversion.

## Case presentation

### Presentation, history, and ocular examination

**Fig. 1:**
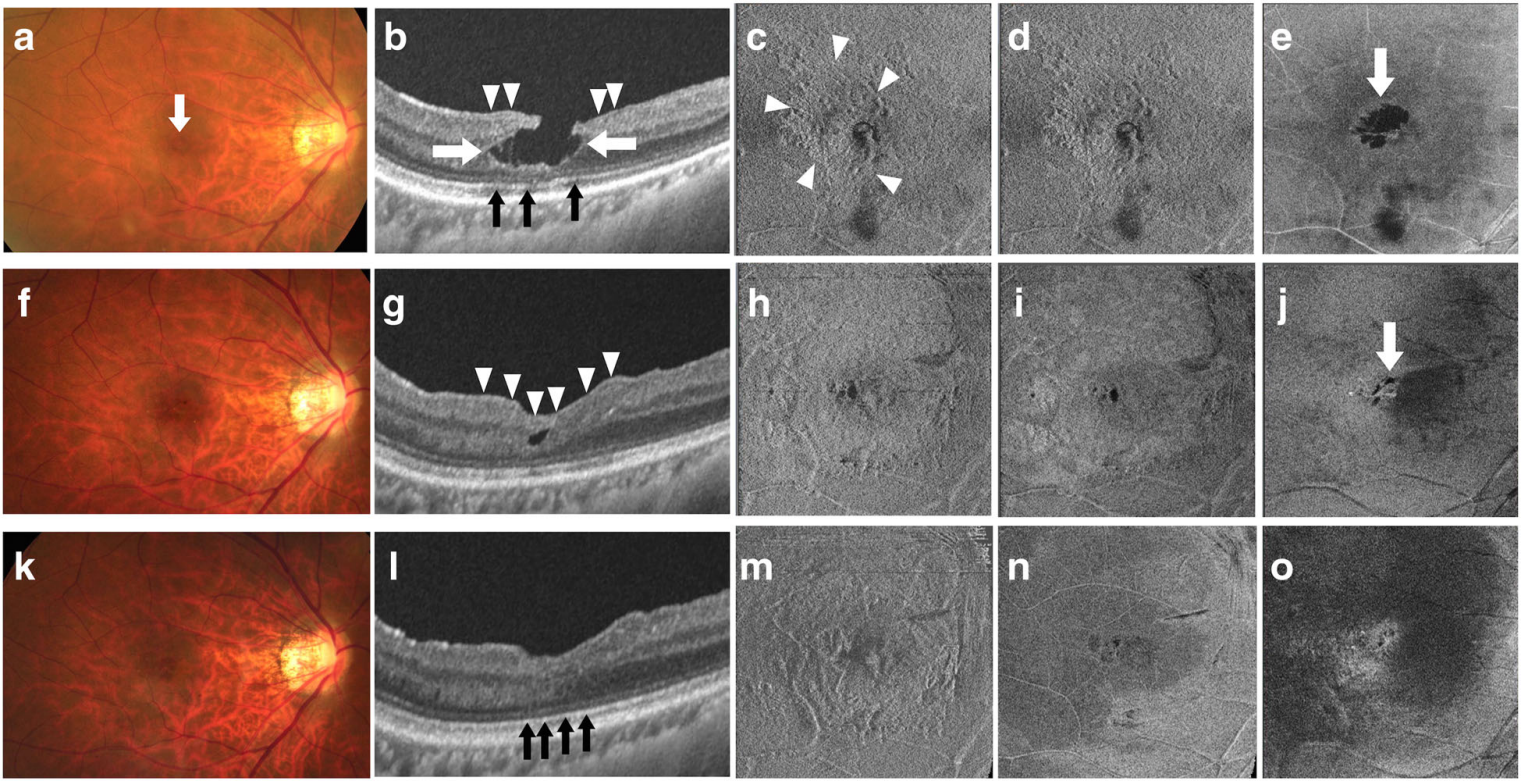
Preoperative and postoperative fundus photographs and OCT images of a 62-year-old man’s right eye. **a–e** preoperative images; **f–j** one month postoperative images; **k–o** three month postoperative images. **a**, **f**, **k** colour fundus photographs; **b**, **g**, **l** B-scan images; **c**, **h**, **m**, en face images at the internal limiting membrane (ILM) level; **d**, **i**, **n** en face images at 10 μm below the ILM level; **e**, **j**, **o** en face images at the outer nuclear layer (ONL) level. At the initial visit the macula was slightly reddish with macular hole-like conformation in the right eye (arrow, **a**). B-scan imaging shows the retinal cleavage (white arrows, **b**) and LHEP (arrowheads, **b**) at the macula. The ellipsoid zone was almost continuous but showed an irregular reflection intensity (black arrows, **b**). En face imaging revealed ERM or LHEP at the level of the ILM (arrowheads, **c**). There was no retinal fold at 10 μm below the ILM level (**d**). There was a retinal cleavage at the ONL level (arrow, **e**). At 1 month after surgery, the retinal cleavage was no longer present (**f** and **g**). B-scan imaging shows the presence of the embedded LHEP and the inverted ILM, although it is difficult to distinguish the two because they seem to be integrated (arrowheads, **g**). At 3 months after surgery, the foveal contour was further improved (**k**–**o**). B-scan imaging shows complete recovery of the ellipsoid zone (black arrows, **l**)

A 62-year-old man was referred to our clinic mainly for central visual disturbance and metamorphopsia in his right eye lasting more than 2 months. The patient had no significant history of systemic disease other than hypertension. At his initial visit, the best corrected visual acuity (BCVA) was 20/29 in his right eye and 20/17 in his left eye. The vertical and horizontal M-CHARTS (Inami & Co., Ltd., Tokyo, Japan) scores were 0.9° and 0.5° in the right eye, respectively. No distortion was detected by M-CHARTS in the left eye. A slit-lamp examination of the anterior segments revealed mild cataracts (grade I according to the Emery-Little classification) in both of his eyes. A fundus examination of his right eye showed a slightly reddish macula with a macular hole-like conformation (Fig. 1a).

### OCT findings

As described in a previous report [9], the morphological features of the macula were identified by utilizing two swept-source OCT (SS-OCT) imaging methods (DRI OCT-1 Atlantis, TOPCON Corporation, Tokyo, Japan): radial B-scan imaging and en face imaging (Fig. 1b–e). The radial B-scan image confirmed the presence of partial-thickness defect of the macula with degenerative retinal cleavage as well as LHEP at the surface of the retina (Fig. 1b). The central retinal thickness was 156 μm, and the ellipsoid zone was almost continuous but showed an irregular reflection intensity. En face imaging revealed a membrane structure on the macula, but no retinal fold was observed (Fig. 1c and d). There was a retinal cleavage from the level of the ILM to the level of the outer nuclear layer (Fig. 1c–e). Based on these findings, we diagnosed the patient as degenerative LMH with LHEP.

### Surgical procedure

**Fig. 2:**
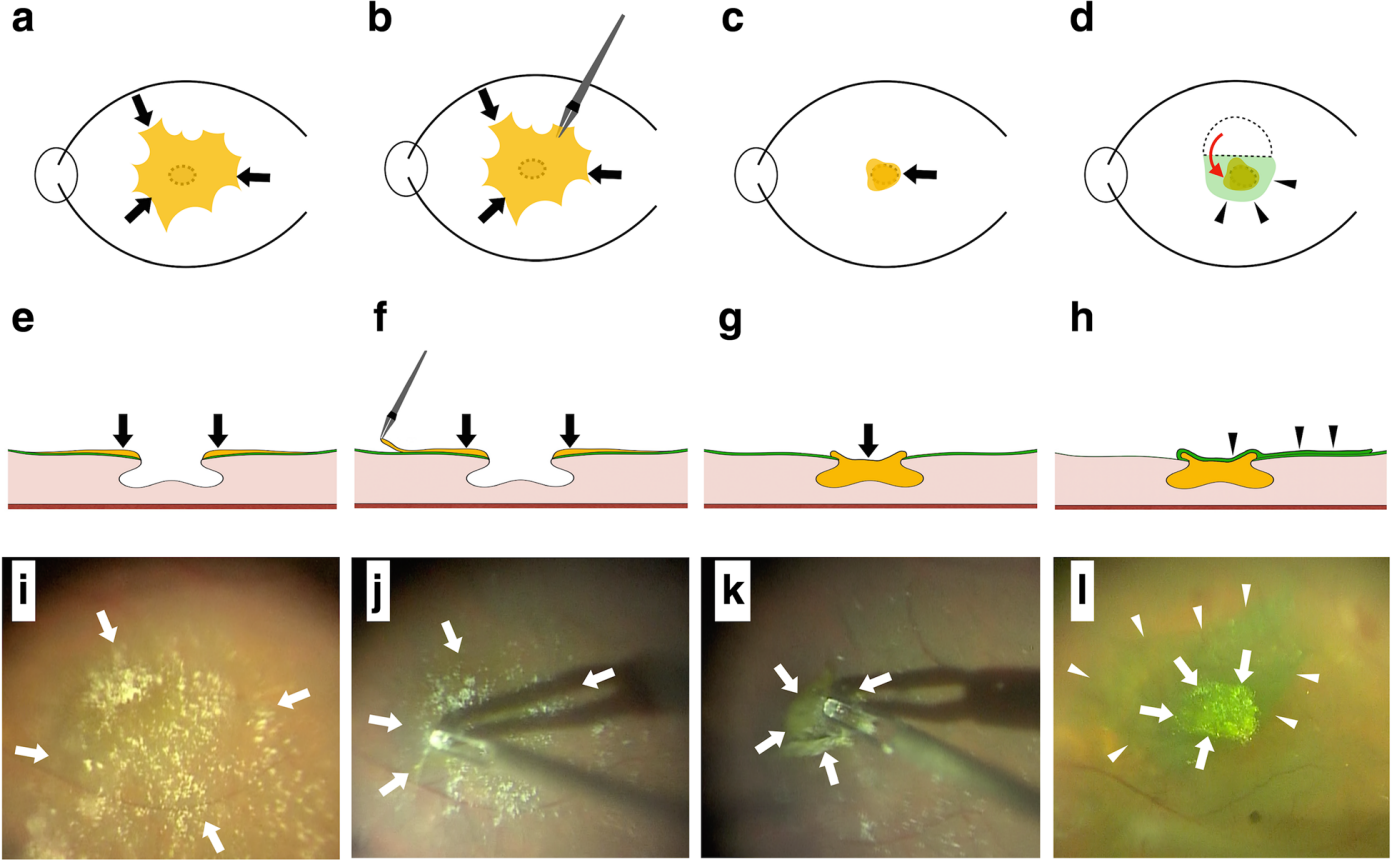
Schematic drawing and intraoperative photographs of embedment of the lamellar hole-associated epiretinal proliferation (LHEP) combined with internal limiting membrane (ILM) inversion for the treatment of lamellar macular hole. Before the operation, LHEP was observed on the surface of the macula (arrows in **a**, **e**, and **i**). The LHEP was peeled centripetally toward the macula with intraocular forceps and left attached to the edge of the LMH (**b**, **f**, and **j**; arrows indicate LHEP). The peeled LHEP was trimmed to fit the size of the retinal cleavage, and the remnant LHEP was embedded into the retinal cleavage (**c**, **g**, and **k**; arrows indicate LHEP). We then inverted the ILM from upper to lower (arrow in **d**) so that the ILM completely covered the LMH with the embedded LHEP (**d**, **h**, and **l**; arrowheads indicate ILM and arrows in **l** indicate LHEP)


To treat the case patient, we performed both LHEP embedment into the retinal cleavage as well as ILM inversion. Briefly, after performing phacoemulsification with intraocular lens implantation and a 25-gauge micro-incision vitrectomy, the LHEP was centripetally peeled off of the retina using intraocular forceps and was left attached to the edge of the LMH (Fig. 2a, b, e, f, i, and j). After trimming the LHEP using a vitreous cutter (Alcon Laboratories, Inc., Fort Worth, Texas) to fit the size of the retinal cleavage, the remnant LHEP was gently massaged centripetally over the LMH and thus embedded into the retinal cleavage (Fig. 2c, g, and k). Next, the ILM was visualized with indocyanine green and then peeled from the periphery towards the LMH. During this peeling, the ILM was not completely removed from the retina but was instead left attached to the edge of the LMH. The ILM was then inverted from upper to lower so that it completely covered the LMH with the embedded LHEP (Fig. 2d, h and l) [21]. During the ILM inversion, 1% low molecular weight hyaluronic acid (Opegan; Santen Pharmaceutical Co. Ltd., Osaka, Japan) was used to control the direction of the ILM flap inversion. Thereafter, sodium hyaluronate-chondroitin sulphate (Viscoat; Alcon Laboratories, Inc., Fort Worth, Texas) was placed on the inverted ILM in order to stabilize the flap. At the end of surgery, fluid-air exchange was performed, and the vitreous cavity was filled with 20% SF_6_ gas. Small amount of these viscoelastic substances (also known as ophthalmic viscosurgical devices) were intentionally left on the inverted ILM flap. The patient remained in the prone position for 3 days after the surgery. [See Additional file 1].

**Additional file 1:** After vitrectomy, the LHEP was centripetally peeled off of the retina using intraocular forceps and was left attached to the edge of the LMH. The LHEP was gently massaged centripetally over the LMH and thus embedded into the retinal cleavage. The ILM was peeled from the periphery towards the LMH but was not completely removed from the retina. The ILM was then inverted from upper to lower so that it completely covered the LMH with the embedded LHEP. During the ILM inversion, 1% low molecular weight hyaluronic acid was used to control the direction of the ILM flap inversion. At the end of surgery, fluid-air exchange was performed, and the vitreous cavity was filled with 20% SF_6_ gas. The patient remained in the prone position for 3 days after surgery. (MP4 24106 kb)


### Post-operative recovery of the macula

One month after the surgery, both B-scan and en face OCT images showed that most of the retinal cleavage had disappeared (Fig. 1f–j). B-scan imaging showed the presence of embedded LHEP and inverted ILM, although it was difficult to distinguish these two from each other because they appeared to be integrated (arrowheads, Fig. 1g). Three months after the surgery, the foveal contour had further improved (Fig. 1k–o), and the ellipsoid zone had recovered (Fig. 1l, arrows). Furthermore, BCVA had improved to 20/20 and the vertical and horizontal M-CHARTS scores in the right eye had improved to 0.8° and 0°, respectively.

## Discussion and conclusion

In this report, we show that combining the LHEP embedding technique with ILM inversion is an effective surgical treatment for degenerative LMH with LHEP. Two main reasons may explain why this combination of surgical techniques is more effective for treating LMH than the LHEP embedding technique alone. The first reason is that inverting the ILM makes it more likely that embedding the LHEP into the retinal cleavage will be successful. In the absence of an inverted ILM, there is a higher possibility that the embedded LHEP will move out of the retinal cleavage during fluid-gas exchange. In contrast, inverting the ILM and placing it over the LHEP can stabilize the LHEP into the retinal cleavage. The second reason this combination of surgical techniques might be more effective is that the inverted ILM might be expected to facilitate the healing effects of the embedded LHEP on the macula. LHEP is mainly composed of glial cells [12], which are thought to play a major role in the healing process of the macula when embedded into the retinal cleavage [14]. Recently, we performed ILM inversion to treat experimental macular hole in a monkey model. We found that the ILM, which is the basement membrane of Müller cells, functioned as a scaffold to promote the proliferation and migration of glial cells. Furthermore, the activated glial cells produced various neurotrophic factors as well as bFGF [20]. Therefore, it is reasonable to suppose that the inverted ILM might have acted as a scaffold of embedded glial cells and facilitated the repair process of the macula by glial cells in the current case.

Recent advances in OCT have revealed that LMH can be classified into two types based on the presence or absence of pathological retinal traction [5, 9]. LMHs that show pathological retinal traction are classified as tractional LMHs, while those that do not are classified as degenerative LMHs. Recently, we conducted an imaging study to ascertain the appearance rate of LHEP in both tractional and degenerative LMH and found that LHEP was only observed in degenerative LMH [9]. Therefore, the adaptation of the LHEP embedding technique described in this report can be applied only to degenerative LMH with LHEP. One important problem is that the appearance rate of LHEP in degenerative LMH is 80%, and no effective treatment for the 20% of degenerative LMHs that lack LHEP has been developed. Indeed, although ERM and ILM peeling to release retinal traction has been reported to be effective for treating tractional LMH, these techniques are not effective for treating degenerative LMH and may even be harmful as they can lead to postoperative macular hole [10–12]. As discussed above, ILM inversion to treat degenerative LMH without LHEP may improve foveal contour and visual function by facilitating the activation of resident glial cells surrounding the LMH. Further study is required to assess the efficacy of ILM inversion for degenerative LMH without LHEP.

There are several limitations to the present report, wherein we describe only one case. The follow-up period is short, and there is a possibility that long-term follow-up will show the recurrence of LHEP or the development of secondary ERM. Furthermore, there is a risk that excessive proliferation of glial cells and inverted ILM may occur, resulting in scar formation at the macula and subsequent visual disturbance.

In conclusion, our case report suggests that combining the LHEP embedding technique with ILM inversion might be an effective treatment for LMH with LHEP. Further prospective studies involving a larger number of patients will be required to determine the actual efficacy of this technique.

## Abbreviations

BCVA: Best corrected visual acuity
ERM: Epiretinal membrane
ILM: Internal limiting membrane
LHEP: Lamellar hole-associated epiretinal proliferation
LMH: Lamellar macular hole
OCT: Optical coherence tomography
ONL: Outer nuclear layer
SS-OCT: Swept-source OCT
